# What Learning Resource Persons do Clinical Medical Students Prefer?

**DOI:** 10.15694/mep.2018.0000158.1

**Published:** 2018-08-03

**Authors:** Anupong Kantiwong, Sakarn Charoensakulchai

**Affiliations:** 1 Phramongkutklao College of Medicine

**Keywords:** Learning Resource Person, Medical Student

## Abstract

This article was migrated. The article was marked as recommended.

Introduction

Nowadays, clinical medical students need to find suitable learning resource persons to cope with knowledge and develop their skills. Resource persons include faculties, residents, peers or each individual medical students’ self-study. The aim of this study was to compare the differences perspectives and give feedback to stakeholders for learning achievements.

Methods

142 medical students answer 5-rating scale questionnaire including 6 aspects; knowledge acquired, accuracy of information, clinical skills, active learning stimulation, comfortable learning environment and time consuming which each had 4 question. Statistical analysis was compared outcomes between groups of resource person by using one-way ANOVA and exploratory factor analysis (EFA).

Results

Simulation of active learning was greatly impacted by faculties (component matrix 0.793) with self-study in the second place (component matrix 0.781). Peers got least impact in acquiring knowledge (component matrix 0.707) but was greatly impacted on clinical skill (component matrix 0.717). Self-study had least impact on clinical skill (component matrix 0.521). Learning with residents and self-study had greatest impact on comfortable learning environment (component matrix 0.813, 0.809 respectively). Peers had highest impact as the learning resource who consumed least time in learning (component matrix 0.858) and accuracy of information (component matrix 0.784). Faculties had lowest impact on accuracy of information (component matrix 0.506). There were significant different average student perspective scores from multiple resources learning (p-value 0.01). In general, students mostly favored learning with residents while faculties got second place; however, there was insignificant differences between peers and self-study.

Conclusion

Residents were the most favored learning resource because they had more experiences and knowledge than peers and had small age gap with students, thus students felt more comfortable to discuss. In addition, faculties, peers and self-study were important as they could still stimulate medical students to develop other important skills (communication skill, clinical skill and stress management).

## Introduction

The generally practiced six-year course of undergraduate medical education curriculum in most medical schools in Thailand is divided into three parts;

• Pre-medical science year which spanned over the first year,
• Pre-clinical science years which covered the second and the third years and
• Clinical science years which included fourth, fifth and sixth years.

After completion of the whole curriculum, medical students would graduate with doctor of medicine degree.

It is especially in the clinical science years setting of which learning shifts from lectured-based that is primarily practiced during pre-clinical years toward patient caring. Learning in clinical years focuses on integrating basic science knowledge into arts of real patients caring. This change of learning method required well adaptive schemes and learning strategies exercised individually by each medical student in order to become successful in clinical years learning.

Usually in Phramongkutklao College of Medicine, as well as other medical schools in Thailand, medical students enrolled in clinical science years frequently spent most time of their days in wards of different departments such as internal medicine, surgery, obstetrics-gynecology, pediatric, psychiatry and etc. During these times, they would spend their time with residents of each departments and peers, either senior or junior. Residents’ role in the ward is mainly service patient caring. The higher authorities in the ward than residents in patient caring are faculties who specialized in their own fields of knowledge. Faculties’ roles are supervisors for residents in patient caring and treatment and being teachers for medical students. Medical students’ major task would be that of learning from residents and faculties’ practices and teachings with additional roles would be assistant and co-worker with residents in patient caring.

This is also a universal trend in context of clinical year learning. Though traditionally faculties are considered a major learning resource person ( Shershneva et al, 2005), there are other source of knowledge in form of persons where medical students can seek. As clinical medical students spent most of their time with residents, a significant portion of their knowledge is attributed to residents’ teaching. (Bing-You and Sproul, 1992). As for residents as a learning resource, it was found that medical students value resident as a resourceful teacher who provide clear and home-taking points ( Melvin et al, 2014). Residents also act as supervisor in clinical skills and give feedbacks to medical students and in addition, medical students learn to be professional by idolize residents’ professional model ( Bordley and Litzelman, 2000 and Busari et al, 2003).

Peers are also considered a learning resource ( Shershneva et al, 2005). It is shown that peers’ teaching can achieve equivalent learning outcomes compared to conventional learning method in undergraduate curriculum ( Yu et al, 2011). Peer-teachers provides learners with knowledge and a better understanding in learners’ difficulties in learning. ( Blanchard, 2015). Also, peers as teachers also enhanced peer-teachers’ teaching skills and clinical knowledge ( Yu et al, 2011 and Blanchard, 2015).

Self-study is also a useful skill for learning medicine which is considered a lifelong learning. It is found that higher readiness for self-study correlates with higher clinical performances ( Shokar et al, 2002). Also, self-study is a major skill employed in problem solving of which is a critical point in patient treatment. These problems stimulate medical students to undergo self-study to find satisfied answers. Stimulating self-study skill will enable medical students’ developments in other fields than knowledge acquired. These developments include cognitive skill, setting objective of learning and learning strategies ( Hmelo-Silver, 2004).

Interaction with various groups of people allows medical students enrolled in clinical science years many opportunities to acquire knowledge, both theoretical and practical, from different learning resource persons. These learning resource persons include faculties, residents and peers. And as mentioned above, self-study is a required method of achieving knowledge, thus, each medical student himself or herself is considered a learning resource person by the way of researching knowledge via self-study. However, each learning resource person might differ from one another in terms of knowledge accord to specialties, experiences, thinking processes and update of knowledge which can affect medical students’ perspectives toward each learning resource persons. In addition, learning environments might also affect how medical students view each learning resource persons.

The purpose of this study are to compare medical students’ perspectives toward each learning resource persons, to explore impact factors of each learning resource persons toward each aspect of learning perspectives.

## Methods

A descriptive cross-sectional study was conducted in October 2017. The subjects in this study was clinical science year medical students. The inclusion criteria included whole clinical science year medical students. The exclusion criteria included sixth year medical students who, by the time this study was being done, were rotating in affiliated teaching hospitals other than Phramongkutklao Hospital, the college’s teaching hospital.

A questionnaire consisting of two parts was developed. The first part was demographic part comprised of three questions. The first question was the year each student was being currently enrolled which included fourth, fifth and sixth years. The second question was grade point average (GPA) which included the ranges of 2.00-2.49 (C), 2.50-2.99 (C+), 3.00-3.49 (B) and 3.50-4.00 (B+ and A). The grades below 1.99 (D+, D and F) were not included in the ranges as those who acquired GPA in this range would be retired. The third question was gender of subjects enrolled.

The second part included a 5-Likert scale rating questionnaire involving perspectives toward learning resource persons. This included six aspects of perspectives - knowledge acquired, accuracy of information, clinical skill, active learning stimulation, comfortable environment and time consuming - toward each group of learning resource person - faculties, residents, peers and self-study. The questionnaire composed of six parts of which were perspectives toward each learning resource persons in the aspect of knowledge acquired, perspectives toward each learning resource persons in the aspect of accuracy of information, perspectives toward each learning resource persons in the aspect of clinical skill, perspectives toward each learning resource persons in the aspect of active learning stimulation, perspectives toward each learning resource persons in the aspect of comfortable environment and perspectives toward each learning resource persons in the aspect of time consuming. The total amount of questions were 24 items.

After the creation, the whole questionnaire was uploaded online to Google Drive. The online questionnaire was sent to all medical students included in this study. After the response was fed back, the data was analyzed via standard statistic program. Mean differences between each perspective toward each learning resource person were compared using one-way ANOVA. We also sought to explore the impact factors each learning resource person yield toward each aspect of perspectives by utilizing exploratory factor analysis (EFA). The higher impact factor would imply that medical students’ votes were more vary in scores, while the lower impact factor implied that the scores were grouped in unvarying cluster.

Furthermore, we randomly interviewed 4 clinical medical students. The purpose of interview was to obtain opinions in order to describe the statistical result of the study.

## Results/Analysis

From the total population of 200 clinical year medical students, 142 responded, counting 71.0% of total population. The majority of subjects were fourth year medical students of which composing 63 out of 142 (44.4%). Male and female medical students shared almost the same proportion of which 73 (51.4%) were from male and 69 (48.6%) were from female. The GPA range which most medical students received was 3.00-3.49 (B) which included 64 (45.1%) medical students. The classified number of medical students who gave response was categorized in years they were enrolling by genders and GPA as displayed in Table 1.

**Table 1.** General characteristics of clinical year medical students of Phramongkutklao College of Medicine in October 2017

Characteristics	Year		
	Fourth N (%)	Fifth N (%)	Sixth N (%)
*Genders*			
Male	32 (22.5%)	30 (21.1%)	11 (7.7%)
Female	31 (21.8%)	29 (20.4%)	9 (6.3%)
*GPA*			
2.00-2.49 (C)	1 (0.7%)	1 (0.7%)	1 (0.7%)
2.50-2.99 (C+)	11 (7.7%)	18 (12.7%)	5 (3.5%)
3.00-3.49 (B)	36 (25.4%)	22 (15.5%)	6 (4.2%)
Above 3.50 (B+ and A)	15 (10.6%)	18 (12.7%)	8 (5.6%)

We also assessed the reliability of our questionnaire by using alpha-coefficient. We used 0.7 as the threshold of reliability of the questionnaire. From the total of 24 items, it yielded the alpha-coefficient at 0.75 which lied in range above 0.7. Thus, our questionnaire was deemed reliable and valid.

From the statistical result, it was found that residents received the highest mean score and was followed by faculties, peers and self-study.

One-way ANOVA analysis showed a significant difference in perspective between 4 groups of learning resource persons with p-value at 0.01 at 95% confidential interval. When compared two learning resource persons it was found that there were significant differences in perspectives between faculties and residents, faculties and peers, faculties and self-study, residents and peers and residents and self-study. However, there was no significant difference in perspectives between peers and self-study. The result was shown in Table 2.

**Table 2.** Analytic result of comparison between medical students’ perspectives toward 4 groups of learning resource persons of Phramongkutklao College of Medicine

Mean				One-way ANOVA					
F	R	P	S	Levene Statistic	P	F	p	Mean difference comparisons	p
22.6	23.8	20.6	20.5	1.6	0.178	40.2	0.001	R > F	0.010
								R > P	0.001
								R > S	0.001
								F > P	0.001
								F > S	0.001
								P > S	1.000

Note:

F = Faculties, R = Residents, P = Peers, S = Self-study Significant level at p-value 0.05 at 95% confidential interval

We further explored the perspectives of medical student from fourth and fifth years separately. The sixth year’s perspectives were not explored as there were too few subjects to be analyzed.

Results from the fourth year medical students showed a significant difference in perspective between 4 groups of learning resource persons with p-value at 0.01 at 95% confidential interval. It happened that residents received the highest mean score from fourth year medical students. As we compared two learning resources, it was found that there were significant differences in perspectives between faculties and peers, faculties and self-study, residents and peers and residents and self-study. There was no significant difference in perspectives between faculties and residents and peers and self-study. The overall result was shown in Table 3.

**Table 3.** Analytic result of comparison between fourth year medical students’ perspectives toward 4 groups of learning resource persons of Phramongkutklao College of Medicine

Mean				One-way ANOVA					
F	R	P	S	Levene Statistic	p	F	p	Mean difference comparisons	p
22.7	23.6	20.2	20.9	0.3	0.797	16.6	0.001	R > F	0.527
								R > P	0.001
								R > S	0.001
								F > P	0.001
								F > S	0.006
								P > S	1.000

Note:

F = Faculties, R = Residents, P = Peers, S = Self-study Significant level at p-value 0.05 at 95% confidential interval

Also, fifth year medical students showed almost similar trend with the fourth years. There was a significant difference in perspective between 4 groups of learning resource persons with p-value at 0.01 at 95% confidential interval. Residents also received highest mean score. There were significant differences in perspective between faculties and residents, faculties and peers, faculties and self-study, residents and peers and residents and self-study. Perspectives toward peers and self-study were not significantly different. The overall result was shown in Table 4.

**Table 4.** Analytic result of comparison between fifth year medical students’ perspectives toward 4 groups of learning resource persons of Phramongkutklao College of Medicine

Mean				One-way ANOVA					
F	R	P	S	Levene Statistic	p	F	p	Mean difference comparisons	p
22.6	24.1	21.0	20.4	1.475	0.222	19.7	0.001	R > F	0.024
								R > P	0.001
								R > S	0.001
								F > P	0.021
								F > S	0.001
								P > S	1.000

Note:

F = Faculties, R = Residents, P = Peers, S = Self-study Significant level at p-value 0.05 at 95% confidential interval

**Figure 1.**
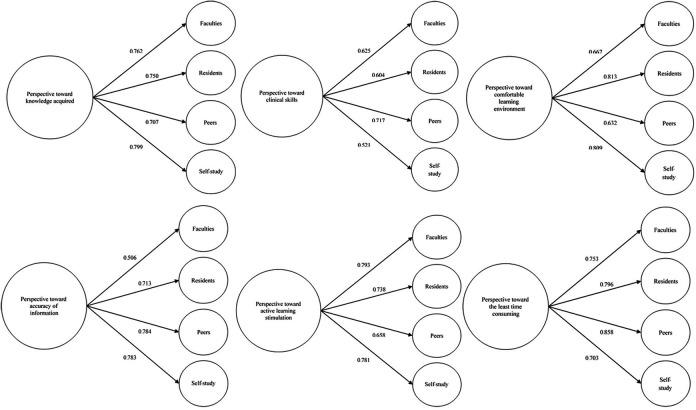
Impact factor of each learning resource person on student perspectives

We further explored impact factors of each learning resource person toward each aspect of perspective which was fully displayed in *Figure 1.* Faculties had highest impact factor on knowledge acquired (component matrix 0.762) and stimulation of active learning (component matrix 0.793). However, they had lowest impact factor on accuracy of information (component matrix.0.506). Residents had highest impact factor on comfortable learning environment (component matrix 0.813) and followed by self-study (component matrix 0.809). Peers had highest impact factor on clinical skills (component matrix 0.717), least time consumed for learning (component matrix 0.858) and accuracy of information (component matrix 0.784). On the other hand, they had the least impact on knowledge acquired (component matrix 0.707). For self-study, it had second highest impact on simulation of active learning (component matrix 0.781) after faculties, but had lowest impact on clinical skills (component matrix 0.521).

## Discussion

As the result had demonstrated, clinical medical students prefer learning with residents. It could be explained by our interview with a focus group of 6 medical students. The reasons behind this result were that medical students and residents had fewer age gap when compared to faculties. Fewer age gap allowed medical students to freely and more comfortably ask questions and discuss with residents more than with faculties. In addition, as stated previously that medical students spent most of their time with residents in the ward, it resulted in close interaction between both groups which made approaching to residents easier than approaching to faculties. All subjects in the focus group agreed that residents also had greater experiences and knowledge than peers and it was easier to learn from the summarized information than summarizing information by themselves by self-study. One subject stated that residents also had more time to care and were closer to patients than faculties, thus it resulted in better real situation when residents teaches medical students.

On the other hand, perspectives from fourth year medical students toward faculties and residents were not significantly different. Two subjects reasoned that as fourth year medical students were ‘new comer’ to the clinical science course, they would collect all clinical knowledge from reliable sources which in this place were faculties and residents. Also, fourth year medical students had the least clinical experiences, thus, they would believe that both faculties and residents could provide information as the same. One subject also reasoned that both faculties and residents teach the basic clinical knowledge which general practitioners are required to know.

It also appeared that perspectives toward peers and self-study were not significantly different. It was explained with the reason that peers’ characteristic and image were not so reliable and not different from each individual medical student. Two subjects explained that both peer-teachers and learner medical students did not have different basic knowledge, as a result, learning from peers would definitely require learner medical students to relearned by themselves which would be self-study. Another reason was that peer-teachers might not have a reliable sources of information. One subject explained that peer-teachers and learner medical students had the same sources of information such as lecture papers provided by faculties, thus there were not much different between peer-teachers and learner medical students’ basic knowledge.

Faculties had low impact factor on perspectives toward accuracy of knowledge. This was because most scores medical students voted were grouped in cluster, making an almost the same direction perspectives, thus lowering its power in making high impact on this aspect of perspective. We examined the result and it showed that most scores were clustered at 5 and an amount in 4 with none in 3, 2 and 1. We took this result into interview with focus group. The subjects answered that this result happened because most medical students tended to believe in information retrieved from faculties because of faculties’ experiences, updated information, specialists in their professional field and their certain role as teacher. This made the questions about accuracy of information from faculties were not in their concern. As a result, the scores for accuracy of knowledge were high on the 5-Likert scale.

Our study also yielded a result that showed an impact factor toward clinical skills which peers had highest impact factor, followed by faculties and the two lowest impact factors owed to residents and self-study respectively. Although both residents and self-study had low impact factors, but their results were drastically contrast to each other. Residents had low impact factor on clinical skill because most students rated score 5 for them, while for self-study, the scores had normal distribution with majority at 3. It could be implied that residents received favor from majority of medical students’ scores in aspect of clinical skills so much that their impact factor had low power. In contrast, self-study had low impact factor on clinical skills because seeing other people with experiences did it first was necessary for learning clinical skills.

Another interesting result showed a high impact factor of peers on the least time consumed. As a result, it could be implied that learning from peer-teachers consumed very least time. The interview with focus group revealed that learning from peers were not much effective when there were plenty of other learning resources available as most of time spent while learning with peers would went through leisurely activities such as chatting topics other than learning, playing mobile games or browsing social networks. Thus, making actual learning time not consumed as it should be. This was linked to the lowest impact factor of peers on knowledge acquired as one of the reasons our focus group provided was that activities other than learning and discussing learning topics would be so frequent that actual learning time decreased.

There are some limitations to our study. Firstly, our study employed electronic questionnaire in order to collect data. It was sent online to fourth, fifth and sixth years’ medical students, together with tight learning and activities schedules of medical students, we were unable to follow or gather and had all clinical years’ medical students answered our questionnaire, resulting in decreased in response rates. Secondly, sixth year medical students were usually assigned to rotate in other affiliated hospitals than Phramongkutklao Hospital, our college’s teaching hospital, thus, it became more difficult to followed and gathered more responses from sixth year medical students. Had they were available, we expected that their experiences and their opportunities to meet and working with other learning resource persons in other hospitals would resulted in a more different point-of-view when compared to fourth and fifth years’ medical students which were only assigned to operate in Phramongkutklao Hospital.

## Conclusion

Finding the suitable learning resources can help students achieve their goal in health care learning. Residents were the most favored learning resource because they had more experiences and knowledge than peers and had small age gap with students, thus students felt more comfortable to discuss. In addition, teachers, peers and SDL were important as they could still stimulate medical students to develop other important skills.

## Take Home Messages

We expected that our study could bring useful information to be utilized. Firstly, residents should be assigned some teaching role to medical students. Residents could be benefited to medical students’ clinical learning because of their close relationship, making learning became easier to approach. In addition, some residents would one day in the future become faculties in medical schools and teaching role would be an inevitable role, thus preparation when they were residents would be crucial.

## Notes On Contributors

**Anupong Kantiwong, MD** (Instructor, *Department of Pharmacology, Phramongkutklao College of Medicine, Bangkok, Thailand)*

**Sakarn Charoensakulchai** (Co-researcher *, Phramongkutklao College of Medicine, Bangkok, Thailand)*
