# Supplementary material for: What Learning Resource Persons do Clinical Medical Students Prefer?
Source: MedEdPublish (2016). 2018 Aug 3;7:158. [Version 1] doi: 10.15694/mep.2018.0000158.1 (PMC10701837; doi:10.15694/mep.2018.0000158.1)
Supplement: Peer review history

# Reviewer report: Reviewer response for version 1

**DOI:** 10.21956/mep.19629.r29217

**Published:** 2018-08-13

**Authors:** Trevor Gibbs

**Affiliations:** 1 AMEE

This review has been migrated. The reviewer awarded 3 stars out of 5 An interesting paper that covers an important topic. The paper was unnecessarily long and slightly detracted from the main issues of the paper which was trying to evaluate the teaching value of various teaching encounters and the "teacher involved. The results were somewhat expected and seemed to be in favour of the near-peer format of learning, which I feel we already value. I was looking for more ideas of how these residents or near-peers are involved in the teaching, particularly in Thailand, given the timing difficulty and the conflict between teaching and service that are frequently encountered. Although I have reservations, I do think that the paper should be read by curriculum developers who are looking at how to address teaching resources constraints.

Reviewer Expertise:

NA

No decision status is available

# Reviewer report: Reviewer response for version 1

**DOI:** 10.21956/mep.19629.r29218

**Published:** 2018-08-05

**Authors:** Sateesh Babu Arja

**Affiliations:** 1 Avalon University School of Medicine

This review has been migrated. The reviewer awarded 4 stars out of 5 I thoroughly enjoyed reading this paper. When I read the title, I thought that I will learn the new information regarding the resource persons for clinical students and very interesting title. This paper is well written and easy to understand. It is very interesting to see how students perceive self-study, peer teachers, residents, and faculty members. Clinical students are always comfortable with residents due to less age gap and they spend more time together. Even some studies have shown that assessment of students by residents gives more useful information than faculty members as residents do more repeated observations of clinical students. This paper is useful for academic administrators and this can be an eye opener for some schools which place their students in clinical attachments without residents. I think residents play a major role in educating and training of medical students.

Reviewer Expertise:

NA

No decision status is available
